# Mineralization Content Alters Osteogenic Responses of Bone Marrow Stromal Cells on Hydroxyapatite/Polycaprolactone Composite Nanofiber Scaffolds

**DOI:** 10.3390/jfb3040776

**Published:** 2012-11-14

**Authors:** Timothy T. Ruckh, Derek A. Carroll, Justin R. Weaver, Ketul C. Popat

**Affiliations:** 1 School of Biomedical Engineering, Colorado State University, Fort Collins, CO 80523, USA; Email: t.ruckh@neu.edu; 2 Department of Mechanical Engineering, Colorado State University, Fort Collins, CO 80523, USA; Email: dac376@cornell.edu; 3 Department of Chemical Engineering, Colorado State University, Fort Collins, CO 80523, USA; Email: jweaver@engr.colostate.edu

**Keywords:** nanofiber, scaffold, bone, hydroxyapatite, gene expression

## Abstract

Synthetic tissue scaffolds have a high potential impact for patients experiencing *osteogenesis imperfecta*. Using electrospinning, tissue scaffolds composed of hydroxyapatite/polycaprolactone (HAp/PCL) composite nanofibers were fabricated with two different HAp concentrations—1% and 10% of the solid scaffold weight. After physico-chemical scaffold characterization, rat bone marrow stromal cells were cultured on the composite scaffolds in maintenance medium and then in osteogenic medium. Quantitative PCR, colorimetric assays, immunofluorescent labeling, and electron microscopy measured osteogenic cell responses to the HAp/PCL scaffolds. In maintenance conditions, both Hap/PCL scaffolds and control scaffolds supported cell colonization through seven days with minor differences. In osteogenic conditions, the 10% HAp scaffolds exhibited significantly increased ALP assay levels at week 3, consistent with previous reports. However, qPCR analysis demonstrated an overall decrease in bone matrix-associated genes on Hap/PCL scaffolds. Osteopontin and osteocalcin immunofluorescent microscopy revealed a trend that both mineralized scaffolds had greater amounts of both proteins, though qPCR results indicated the opposite trend for osteopontin. Additionally, type I collagen expression decreased on HAp scaffolds. These results indicate that cells are sensitive to minor changes in mineral content within nanofibers, even at just 1% w/w, and elucidating the sensing mechanism may lead to optimized osteogenic scaffold designs.

## 1. Introduction

Bone tissue engineering remains a highly active area of research largely because of complications associated with challenging healing scenarios [1]. Synthetic tissue scaffolds for bone regeneration have taken several design approaches with the intent of enhancing bone formation by marrow stromal cells (MSCs) that migrate to the injury site. MSCs contain a heterogeneous cell population within which exist progenitor cells capable of differentiating into multiple phenotypes including osteoblasts [2,3,4,5]. In order to direct progenitor cell differentiation, biomimetic designs have aimed to create conditions and signals that would be found in actively remodeling bone tissue. Soluble signals such as growth factors or cytokines have been shown to enhance osteoblastogenesis [6,7,8,9] by activating cell surface receptors. Additionally, immobilizing and presenting extracellular matrix (ECM) proteins such as fibronectin and collagen or short adhesion peptides such as RGS have been shown to enhance proliferation and phenotypic behaviors of MSCs [10,11]. Micro- and nanotopographies have also been shown to modulate cell behaviors on metallic [12,13,14] as well as polymeric implant materials [15]. Previous studies have shown modestly enhanced osteoblast phenotypic behaviors by incorporating hydroxyapatite (HAp), a major constituent of the bone matrix, or other calcium phosphate phases in the bulk of the scaffolds [16,17]. To date, only one investigation with polymer-mineral composite nanofibers has described osteoblast behaviors beyond ALP enzymatic activity and Ca-P neo-mineralization [17,18,19].

Electrospinning has become very popular technique within the tissue engineering community because it can consistently produce polymer fiber scaffolds with diameters ranging from less than 100 nm up to several microns [20]. Under a high electric potential, a Taylor cone of polymer solution forms at the catheter tip. Once the electrostatic forces overcome the viscosity and surface tension, a polymer jet is ejected and it travels from the catheter to a grounded collector positioned nearby with a trajectory that follows the electric potential field lines. As the jet travels, the polymer solvent evaporates, leading to the deposition of very fine fibers on the grounded collector [20,21,22]. In this study, we have fabricated hydroxyapatite/polycaprolactone (HAp/PCL) composite nanofiber scaffolds by electrospinning to examine key changes in osteoblast phenotypic behavior. PCL is a semi-crystalline, biocompatible, aliphatic polyester [23] that has been frequently utilized for tissue engineering applications over the past 10–15 years. It is biodegradable, with a relatively slow degradation rate [24], and its non-acidic degradation pathways add to its appeal in biological applications [25]. As a result, it is generally regarded as a compatible scaffold material for both hard and soft tissues [16,26]. 

Hydroxyapatite (Ca_5_(PO_4_)_3_OH) (HAp) is the primary inorganic phase of bone tissue, and in Haversian bone it resides in gaps at the ends of type I collagen fibrils with a well-controlled crystallographic orientation [27]. As new bone is forming and existing bone is remodeling, osteoblasts secrete bone matrix vesicles (BMVs) containing Ca^2+^-rich fluid and phosphatases such as alkaline phosphatase (ALP) [28,29]. HAp integration into type I collagen comprises a bone subunit, and mature bone tissue is further strengthened by its hierarchical architecture [30,31,32]. Cells are capable of binding to collagen through several integrin heterodimers [33], but binding to HAp crystals is believed to require adaptive proteins, notably osteopontin [34,35,36]. Because HAp is an important component of natural bone tissue, it is an attractive design feature for synthetic bone tissue scaffolds as a means of more closely mimicking the natural tissue composition [17,18,37,38,39,40]. Thus, using the electrospinning technique, we have fabricated HAp/PCL composite nanofiber scaffolds with varying amounts of HAp.

The overall aim of this study was to determine the effect of HAp concentration in randomly oriented HAp/PCL nanofibers on key osteoblast behaviors and bone matrix production. PCL nanofiber scaffolds with two different concentrations of HAp nanoparticles were fabricated by the electrospinning process, thus creating HAp/PCL composite nanofibers. The HAp nanoparticles are attractive because they can be incorporated into the nanofiber architecture during electrospinning without disrupting the nanofibrous architecture. In order to account for the effects of electrospinning on PCL properties and nanoarchitecture on cells, control scaffolds were fabricated without any HAp nanoparticles under similar conditions as the HAp/PCL composite scaffolds. The effects of different HAp concentrations in HAp/PCL composite scaffolds were examined with MSCs cultured in maintenance media through 7 days, and then in osteogenic media for 3 weeks.

## 2. Results and Discussion

### 2.1. Fabrication and Characterization of HAp/PCL Composite Nanofiber Scaffolds

**Figure 1:**
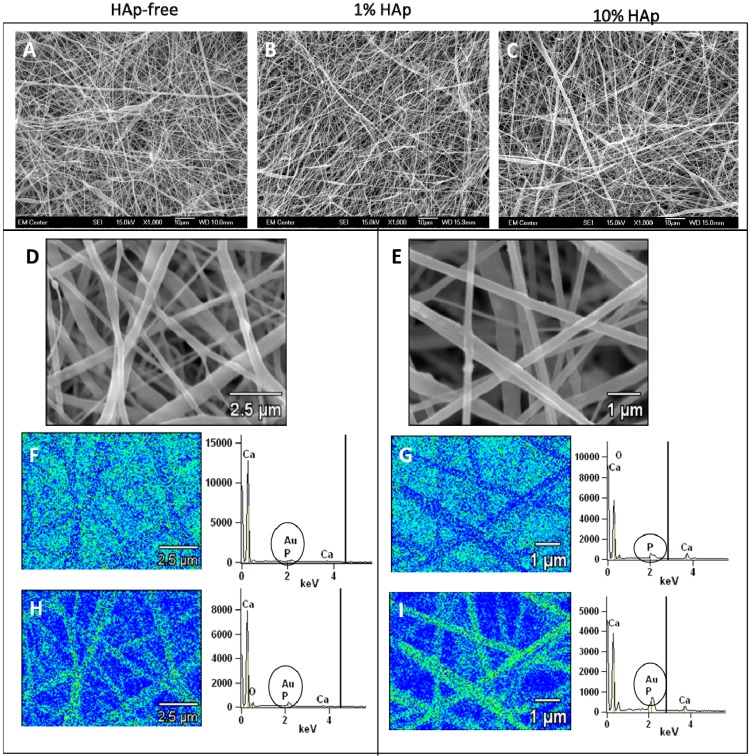
SEM (**A–E**) and EDX (**F–I**) images and spectra for PCL scaffolds with 0% (**A**) 1% (**B**, **D**, **F**, **H**) or 10% (**C**, **E**, **G**, **I**) HAp nanoparticles. Top image is SEM, middle image is EDX spectral image of non-surface nanofibers (no Au coating), and bottom image is EDX spectral image of surface nanofibers (Au coating). All images were taken at 1000× magnification.

**Figure 2:**
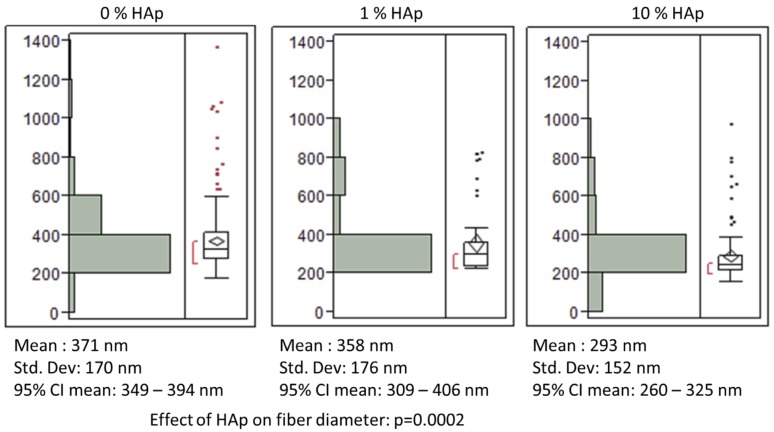
Fiber diameter histogram showing the scatter for measured fiber diameters, the 95% confidence intervals, mean fiber diameter values, and standard deviations for each of the three scaffolds.

This study aimed to determine the effects of HAp nanoparticles in polymer-mineral composite nanofiber scaffolds for bone regeneration. In order to assess the effects of HAp nanoparticles, scaffolds were fabricated with 1% or 10% w/w HAp nanoparticles, and HAp-free scaffolds were used as a control treatment. Prior research demonstrated that HAp nanoparticles can integrate into polymer nanofibers; however studies that did not use a surfactant reported HAp particle agglomeration [19]. Similar prior research with polyester/hydroxyapatite composite nanofibers has also shown that adding hydroxyapatite nanoparticles to the electrospinning solution does not have a significant effect on water contact angle [41]. To prevent agglomeration of hydrophilic HAp nanoparticles in the electrospinning solution with organic solvents and hydrophobic PCL, oleic acid (OLA) was used as a surfactant. Low-magnification SEM images showed no large-scale HAp particle agglomeration, and high-magnification images with EDX spectral scans verified that HAp particles are dispersed randomly within PCL nanofibers in both 1% HAp and 10% HAp scaffolds (Figure 1A–C). There was a small, measurable decrease in the mean fiber diameter for scaffolds with 10% HAp (Figure 2), from 371 to 293 nm. However, for all three scaffold types, >80% of the fibers measured within 200–600 nm in diameter (Figure 2). EDX spectral maps for both types of HAp/PCL composite scaffolds show a difference between the signal from gold-coated surface fibers (Figure 1D and E, bottom images and spectra for each) and the signal from the uncoated interior fibers (Figure 1D and E, top images and spectra for each). Both spectral maps show the presence of calcium and phosphorus, but only the surface spectra show gold. Additionally, the homogenous appearance of spectral maps support the observation that HAp nanoparticles did not agglomerate and cause breaks in the polymer nanofibers. 

Previously, 12-hydroxystearic acid was effective in preventing HAp agglomeration in polymer-mineral composite nanofiber scaffolds [42]. This approach of adding a fatty acid may contribute to the consistency of fiber diameter characteristics between scaffolds with varying HAp concentrations. It is important to note that although the mode was consistent across all three scaffolds, the population characteristics were different and are reflected in the 95% confidence intervals and in the overall main effect being highly significant (Figure 2). In this study, we chose OLA because it is non-toxic and may serve as an additional osteogenic design factor by activating fatty acid receptors. It is a known agonist to peroxisome proliferator–activator receptors (PPARs), a class of nuclear receptors [43,44,45]. PPARs affect transcriptional regulation for a wide range of cellular functions and processes, and agonists for the isoform PPAR have been shown to enhance osteoblastogenesis markers [46,47,48,49]. In order to account for possible effects of OLA as a PPAR agonist, the concentration as held constant for all scaffolds.

**Figure 3:**
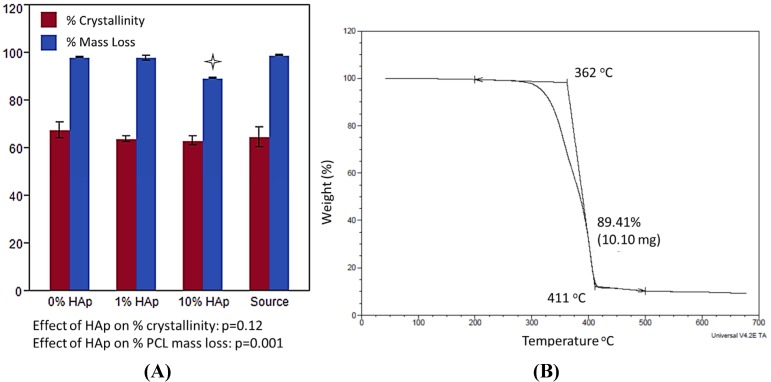
Thermal characterization with (**A**) the % crystallinity and (**B**) mass loss measured by DSC and Thermogravimetric analysis (TGA), respectively. Each treatment (0, 1, or 10% HAp) was measured as well as the source PCL, which was not processed by electrospinning. A 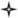 indicates statistically significant difference (p_crit_ = 0.05).

Thermal gravimetric analysis (TGA) and digital scanning calorimetry (DSC) were used to measure the composition and crystallinity of the HAp/PCL composite nanofiber scaffolds. Source PCL (not electrospun) was included to detect any changes to the polymer crystallinity due to the electrospinning process and/or HAp nanoparticles. DSC results (Figure 3A) showed that HAp did not significantly change PCL crystallinity. The percent mass loss due to PCL degradation measured by TGA was significant different between 10% HAp scaffolds and all other samples (Figure 3B), and that difference was approximately 10% of the sample mass. The differences between 1% HAp and 0% HAp (control) scaffolds were too small to be statistically significant in a t-test, although the ANOVA model determined that the effect of HAp on % mass loss was highly significant (p = 0.001). The differences in composition between HAp-free and 1% HAp scaffolds were too small to produce statistical significance, but both scaffolds were significantly different from 10% HAp scaffolds, and EDX confirmed the presence of Ca-P in the 1% HAp scaffolds.

Taken together, the SEM/EDX images combined with TGA and DSC data provided strong evidence that HAp nanoparticles are smoothly incorporated into the PCL nanofibers at the desired concentrations of 1% and 10% w/w. The statistically significant differences in fiber diameter measured by SEM represented a change of ~80 nm (Figure 2), and the fiber diameter histograms showed that all three scaffolds have >80% of their fibers in the same diameter range of 200–400 nm. These differences were within a range of fiber diameters that have been shown to have negligible influences on osteoblasts as well as fibroblasts, a phenotype sharing a very similar transcriptome to that of osteoblasts [50], cultured on nanostructured polymer materials [51,52]. Due to the relatively small changes to fiber diameter and the relative insensitivity of these cells to those small changes, it is reasonable to assume that the differences in fiber diameter are unlikely to influence the cellular responses. Any differences in cell behaviors between the scaffolds are more likely a consequence of variations in HAp concentration.

### 2.2. MSC Response to HAp/PCL Composite Nanofiber Scaffolds in Maintenance Conditions

**Figure 4:**
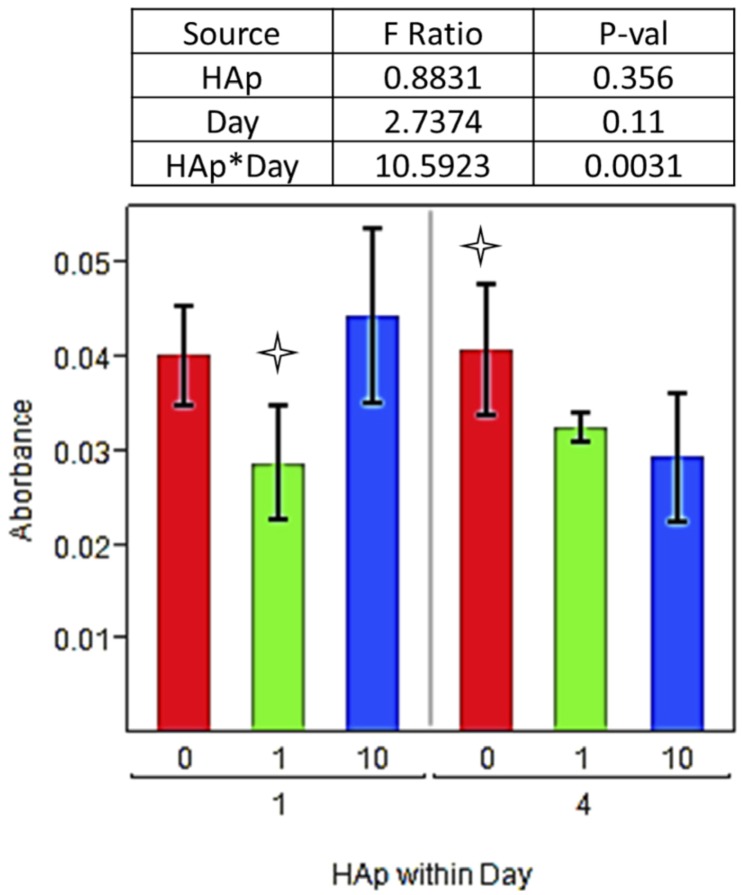
(3-[4,5-dimethylthiazol-2-yl]-2,5-diphenyltetrazolium bromide (MTT) assay values for days 1 and 4. MTT values measured on each scaffold (0, 1, or 10% HAp) are grouped within each day. Bar graph shows daily data, with a 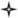 indicating a statistically significant difference (p_crit_ = 0.05) from other treatments within the timepoint. An ANOVA analysis is summarized with F-statistics and p-values for the effects of HAp, day, and the interaction of HAp and day.

Fracture repair entails multiple overlapping healing phases involving wide range of processes for cell recruitment and signaling [53,54,55], ECM production [56,57,58,59], and then continual ECM remodeling [60,61,62]. Fundamental to each of these processes is the ability for cells to adhere and proliferate in the repair area. Calcein AM is a cell-permeable fluorescent stain that is only retained by live cells when esterases cleave the AM group, leaving the impermeable calcein molecule in the cytoplasm. By imaging live cells with calcein AM fluorescent stain, measuring their coverage area, and measuring metabolic activity with an MTT assay, cell adhesion and colonization can be evaluated. For the first seven days after seeding fresh rat bone marrow stromal cells onto scaffolds the cells were cultured in maintenance media free of differentiation factors to allow for the heterogeneous marrow stromal population to populate the scaffold. An MTT assay measured the overall metabolic activity on PCL/HAp scaffolds, and he overall effect of scaffold HAp content was not significant (p = 0.356). However, the interaction effect of Hap*day, meaning the change on each scaffold between days, was highly significant (p = 0.0031), with 10% HAp scaffolds causing a decrease between days one and four and 0% and 1% scaffolds causing very small changes in metabolic activity (Figure 4). 

**Figure 5:**
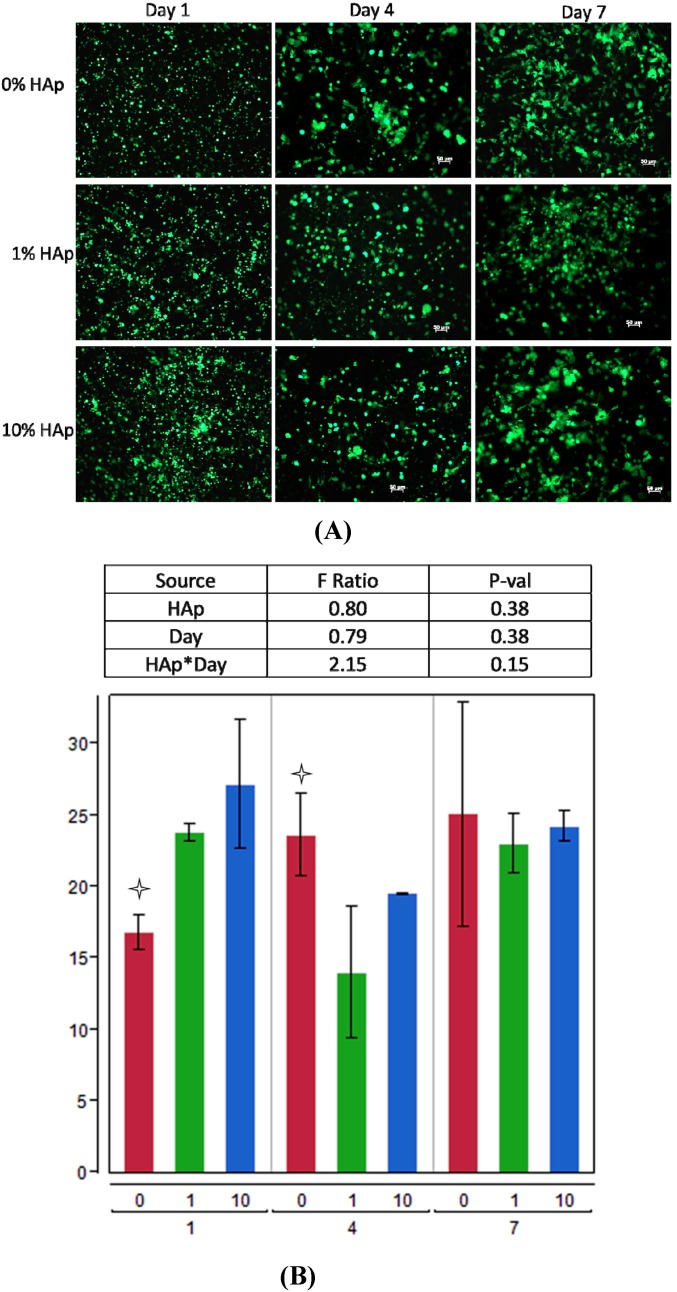
Fluorescent images (**A**) of live cells stained with Calcein AM on HAp-free (0% HAp), 1% HAp, and 10% HAp scaffolds at days 1, 4, and 7 post-seeding. Zoom = 10×, scale bar = 50 mm on each image; Quantitative cell coverage data (**B**) from calcein AM fluorescent images is grouped so that each treatment (0, 1, or 10% HAp) is contained within the day (1, 4, or 7) on which the measurements were taken. Bar graph shows daily data, with a 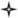 indicating statistically significant difference (p_crit_ = 0.05) from other treatments. An ANOVA summary is presented with F-statistics and p-values for the effects of HAp, time (day), and the interaction of HAp and time.

Metabolic expenditures during the first days of cell seeding are likely to go towards cell proliferation, though the fluorescence microscopy images (Figure 5) demonstrate that substantial cell spreading occurred (cytoskeletal formation and reorganization) during the first week. The ANOVA of cell coverage, calculated from calcein-AM live cell images (Figure 5) shows that HAp also did not significant affect (p = 0.38) cell coverage, although individual t-tests within days one and four revealed that cells on 0% HAp scaffolds were significant different from cells on scaffolds containing either 1% or 10% HAp. Although 0% HAp scaffolds supported the lowest cell coverage on day 1, the MTT assay measure a relatively high level of metabolic activity, which may explain why those scaffolds supported significantly greater cell coverage on day 4. More importantly though, after seven days in culture, the cells on all three scaffolds had densely populated the scaffolds’ surfaces, and there were no significant differences in cell coverage.

Although there were differences in both MTT and cell coverage at days one and four, cell coverage was nearly equal by day seven and the overall effect calculated by the ANOVA model do not meet significance at a 95% confidence level. Thus we reason that the cell populations were very similar at day seven, when differentiation factors were added to the culture. This is consistent with recent literature examining nanofiber scaffolds containing HAp nanoparticles [18].

### 2.3. MSC Differentiation on HAp/PCL Composite Nanofiber Scaffolds

Having established similar scaffold morphologies and similar MSC populations on all three scaffolds, the primary aim of evaluating osteogenic behaviors could be addressed. In order to evaluate the effects of Hap concentration on MSCs in osteogenic medium, a normalized colorimetric ALP assay along with normalized qPCR for five key genes were used. After three weeks in osteogenic medium, cells were also immunolabeled for two bone matrix proteins, OP and OC. 

**Figure 6:**
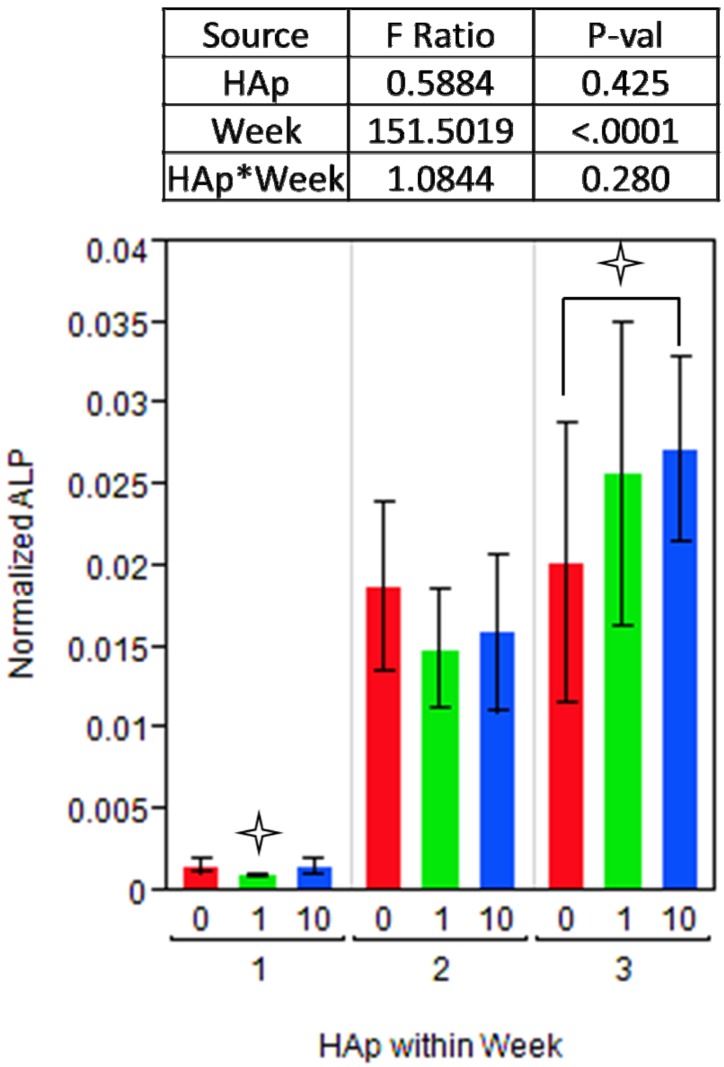
Intracellular alkaline phosphatase (ALP) activity measured by colorimetric assay at weeks 1, 2 and 3. Each measurement was normalized for total protein, as assayed by the BCA method. Bar graph shows data with each HAp level (0, 1, or 10%) grouped within the week (1, 2, or 3) when the measurement was taken. A 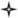 indicates a statistically significant (p_crit_=0.05) difference from other treatments. An ANOVA analysis is presented with F-statistics and p-values for the effects of HAp, week, and the interaction of HAp and week.

Alkaline phosphatase (ALP) is a key enzyme in bone matrix vesicles that cleaves organic phosphate esters, thus supplying mineral nucleation sites with free phosphate ions [28,63,64]. Its expression profile is typically associated with a peak during early differentiation of progenitor cells into immature osteoblast phenotypes, and then production tapers off as osteoblast cells either mature into osteocytes or undergo apoptosis [65]. As that maturation occurs, osteocalcin (OC) expression typically increases, indicating a late-osteoblast state. The ANOVA model for ALP determined that the overall effect of HAp was not significant (p = 0.425), although the effect of changes between weeks was highly significant (p < 0.0001). The dynamic nature of ALP activity in immature osteoblasts may have dominated the ANOVA model. Normalized intracellular ALP activity on all three scaffolds after one week of culture was much lower than the following two weeks (Figure 6). After two weeks in osteogenic conditions, all three scaffolds supported substantial increases in ALP activity, although there were no significant differences within the time point. After three weeks of culture, the cells on 10% HAp produced significantly more ALP than the cells on HAp-free scaffolds, however, neither level was significantly different from 1% HAp scaffolds.

The final time point, after three weeks, shows that ALP is most abundant on 10% HAp scaffolds, and the difference between ALP levels on 10% HAp and HAp-free scaffolds was significant. With ALP activity still high, and qPCR showing that OC expression levels were steady, this suggests that osteoblasts are still relatively immature after three weeks. This may be a consequence of the nanofiber morphology, as our previous work showed prolonged ALP activity on nanofiber scaffolds relative to smooth surfaces in the same culture conditions [15]. 

With regards to the modest increase in ALP activity on polymer-mineral composite scaffolds, other studies using different cell populations (hFOB or human MSCs) also found a similar trend of small increases in ALP activity on mineralized scaffolds [17,18,66,67]. Since these studies used different cell populations and a variety of mineralization levels but yielded similar results, this nominal increase in ALP activity through three weeks on synthetic HAp/PCL composite nanofiber scaffolds can be considered an acceptable and expectable result for *in vitro* studies. 

**Figure 7:**
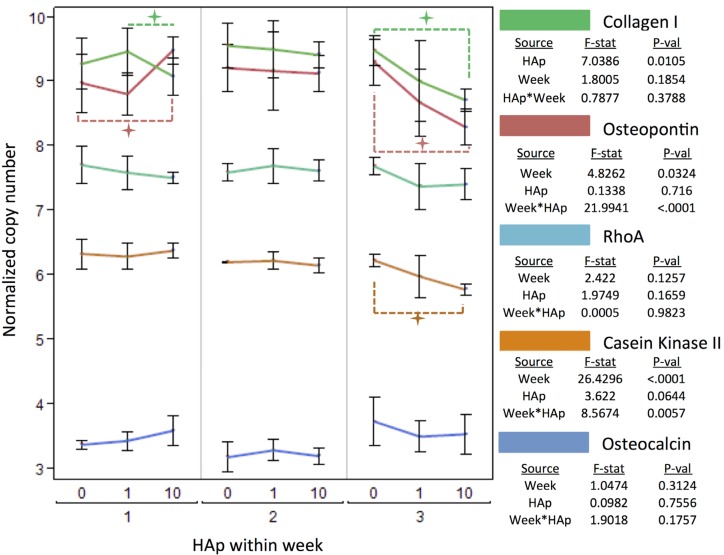
Normalized Collagen I (green), Osteopontin (red), RhoA (light blue), CKII (orange) and Osteocalcin (dark blue) expression measured by qPCR at weeks 1, 2, and 3. Each HAp level (0, 1, or 10%) is grouped *within* a week (1, 2, or 3) so that comparisons are made between treatments and within time points. Each gene was normalized with respect to the copies of RPL13A. A 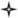 indicates a statistically significance (p_crit_ =0.05) difference from another treatment within the time point. An ANOVA adjusted for multiple comparisons analyzed the effects of HAp, week, and the interaction of HAp by week, and the F-statistics and p-values are summarized for each gene separately.

Quantitative polymerase chain reaction (qPCR) was used to precisely measure the levels of gene expression for five key genes related to osteoblast behaviors: osteopontin (OP), osteocalcin (OC), type I collagen (ColI), RhoA, and casein kinase II (CKII). The expression level for each gene was normalized with respect to RPL13A, a housekeeping gene that encodes for the 60 s ribosomal subunit protein, L13A. Three of the five genes in the qPCR analysis were differentially expressed at significant levels due to HAp concentration within PCL scaffolds—OP (p = 0.0324), CKII (p < 0.001), and ColI (p = 0.0105). Of these, OP ^34,43,48^ and ColI are bone matrix proteins while CKII is the enzyme believed to be primarily responsible for phosphorylating OP [68,69,70]. For all three genes, the overall effect was a decrease in expression when HAp concentration was increased in scaffolds which was largely driven by differences in week three (Figure 7). 

Collagen I (ColI) and osteopontin (OP) are both organic components of bone tissue. Both proteins are expressed by osteoprogenitors, pre-osteoblasts and osteoblasts, though OP is expressed in high levels across multiple phases of differentiation. ColI expression is similar to ALP in that they are both expressed highest by matrix-producing osteoblasts and then down-regulated as the cells mature into osteocytes [71]. In bone tissue, ColI comprises the major organic phase of bone, making up roughly 30% of bone mass, and it also provides integrin binding sites for cells. Bone matrix proteins such as OP, osteonectin (ON), and osteocalcin (OC) all play roles in regulating the manner with which ColI is integrated with HAp crystals in natural bone matrix [72,73,74]. 

OP is part of the family of non-collagenous proteins (NCPs) referred to as SIBLING (Small Integrin-Binding LIgand, N-Linked Glycoprotein) proteins [75]. It contains approximately 30 serine residues, depending on the species, and the degree of phosphorylation has been shown to regulate the exposure or obstruction of specific integrin binding sequences [34,35]. Like ColI, the main effect of HAp on OP expression levels was a significant (p = 0.0324) overall decrease in expression. Using this overall, main effect to measure OP (and also ColI) is meaningful because both proteins would be synthesized and then secreted into the extracellular matrix. Thus the overall expression is a representation of the anticipated accumulation of either protein. 

**Figure 8:**
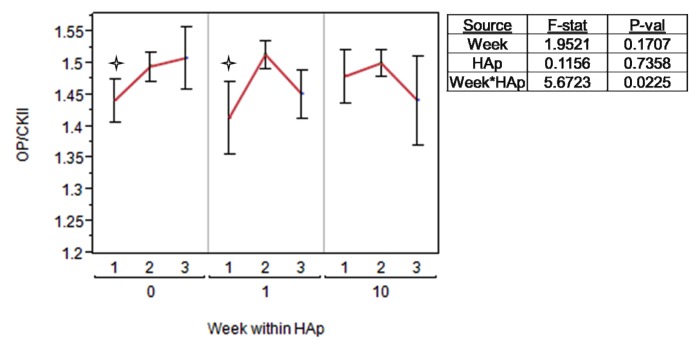
The ratio of osteopontin (OP):CKII calculated and analyzed in an ANOVA. Each HAp level (0, 1, or 10%) is grouped *within* a week (1, 2, or 3) so that comparisons are made between treatments and within time points. A 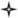 indicates a statistically significance (p_crit_ = 0.05) difference from another treatment within the time point.

Casein Kinase II (CKII) is an intracellular serine/threonine kinase that is believed to be responsible for most of the physiologically relevant phosphorylation for bone phosphoproteins such as OP [68]. It phosphorylates serine residues, and the degree of phosphorylation of bone matrix proteins such as OP is believed to up or down-regulate local bone remodeling behavior by osteoblasts and osteoclasts [35,76]. Differences in OP phosphorylation would potentially be a cellular adaptation to changes in scaffold HAp concentration. The relative OP:CKII the expression ratio does move significantly differently between weeks (Figure 8). The differences are quite small, ranging from 1.4 to 1.5, but OP phosphorylation and the amount of OP present in the extracellular environment are believed to be potent contributors to controlling of neo-mineralization [34,35,68,77,78,79,80,81]. These results do not deliver a definite conclusion on the point, but they are worth noting. The unexpected results from qPCR were that ColI, OP, and CKII expression levels decreased in response to increasing the HAp content in nanofiber scaffolds except for one point, week one on 10% HAp scaffolds. A duplicate trial with a separate cell source produced the same trend, which validated this finding. 

Because OP is a target substrate for CKII, the ratio of OP: CKII was also calculated and analyzed in an ANOVA, and only the interaction effect of HAp*week was significant (p = 0.0225). Figure 8 illustrates this parameter’s physical meaning, showing a general increase in OP:CKII on 0% HAp scaffolds from week one to week three. Conversely cells on 1% HAp scaffolds peaked at week their OP:CKII expression at week two before showing a decrease, and cells on 10% HAp scaffolds were relatively constant in their OP:CKII expression levels. 

RhoA and osteocalcin were both expressed at statistically similar levels on control (0% HAp) and mineralized (1% or 10% HAp) scaffolds (Figure 7). Even within each time point, there were no significant differences measured by a t-test, which supports the observation that these genes were not significantly affected by the concentration of HAp within PCL scaffolds. RhoA is a small GTPase that is responsible for actin stress fiber formation. Recent findings have identified RhoA as not only a regulator of osteoblastogenesis [82,83], but also of mechanotransduction [84] and apoptosis specifically for osteoblasts [85]. It is important to note that RhoA is dependent on phosphorylation by GTPase Activating Proteins (GAPs) in order to become active, and then it is dephosphorylated by Guanine Nucleotide Exchange Factors (GEFs) to return to the inactive state [84]. The qPCR data does not indicate the state of phosphorylation for RhoA. However, RhoA is subject to proteosomal degradation through ubiquitination [86], and osteoblasts have been shown to completely eliminate cytosolic RhoA in as little as 18 hours [87]. Thus, the qPCR data shows that the MSCs on all three scaffolds maintained similar levels of RhoA, which suggests that mechanotransduction was not responsible for differences in osteoblast behaviors. However, this will be a behavior to investigate further in the future.

**Figure 9:**
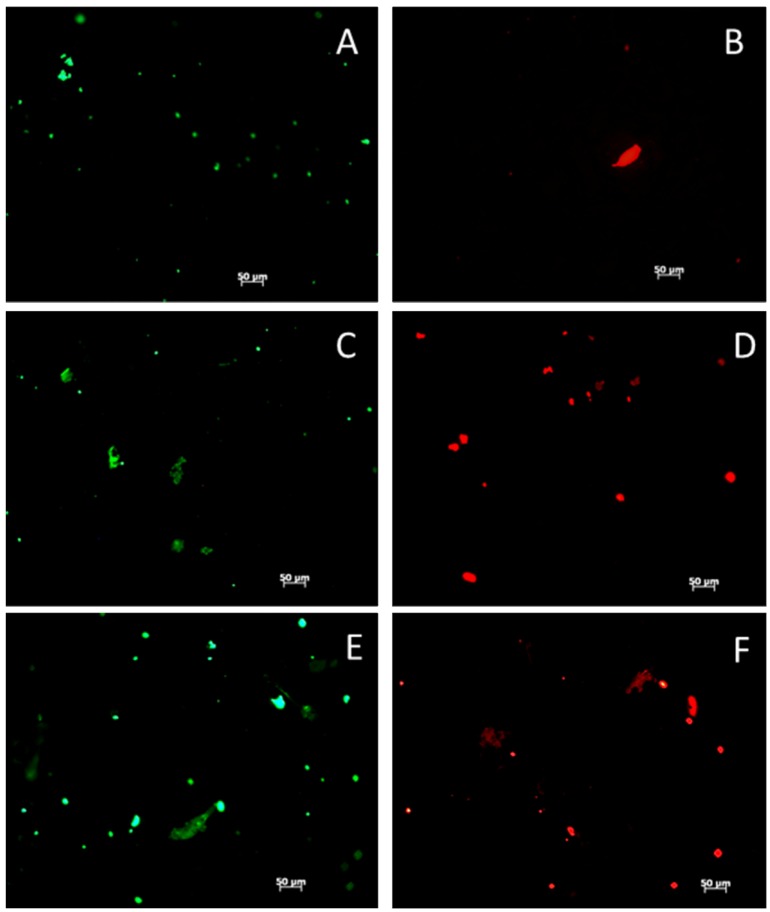
Immunofluorescent images showing the deposition of osteocalcin (green, FITC) and osteopontin (red, TRITC) on nanofiber scaffold surfaces. Images obtained on 0% HAp (**A** & **B**), 1% HAp (**C** & **D**), or 10% HAp (**E** & **F**) scaffolds. Magnification is 10×, scale bar is 50 mm in all images.

After three weeks of culture in osteogenic media, the cells were immunolabeled for either osteopontin or osteocalcin and then viewed under a fluorescence microscope. The images revealed presence of both bone matrix proteins on all of the scaffolds (Figure 9). Many small OC deposits were visible on the scaffold surfaces for control and mineralized scaffolds, though 10% HAp scaffolds appeared to contain the most OC. However, for OP, there were more frequent and larger aggregates (>50 m) observed on 10% HAp and 1% HAp scaffolds than on HAp-free scaffolds. 

The results presented here are similar to the results from another recent study which compared electrospun nanofiber scaffolds with 0%, 9% or 28% w/w HAp nanoparticles in poly(lactic acid) (PLA). In that case, cells on 28% HAp scaffolds outperformed cells on 9% HAp scaffolds for measured ALP and osteocalcin expression measured by qPCR [18]. However, the PCR data presented within that study examined only one time point, rather than the three time points presented herein. In fact, this is the first study to report time-course gene expression data for polymer-mineral composites for bone regeneration. 

The observation that MSCs are sensitive at to HAp concentrations as low as 1% w/w of the scaffold, and that changing between 1% and 10% w/w HAp content elicits different cell responses, are two important findings. The next logical and important step will be to determine the mechanism(s) by which MSCs sense HAp. Furthermore, these studies could examine the potential of HAp/polymer scaffolds to direct stem cell populations towards osteogenic behaviors in the absence of glucocorticoids by performing time-course cytometric population characterization to capture the differentiation of MSCs into mature phenotypes [88]. Changes in the mechanical compliance of the PCL-HAp scaffolds may occur with 10% w/w HAp, and compliance has been shown to be transduced through the RhoA-ROCK-MAPK pathway [84]. However, the manner with which MSCs sense changes in extracellular Ca-P mineralization is not yet clear, and this will need to be explored in the future by focusing on mechanisms by which cells sense the extracellular chemistry (mineralization) and mechanical environment.

## 3. Experimental Section

### 3.1. Fabrication and Characterization of HAp/PCL Composite Nanofiber Scaffolds

Nanofiber scaffolds were fabricated using electrospinning process. A polymer solution was prepared by dissolving of 12% w/v PCL (Sigma) with 3% oleic acid sodium salt in a solvent mixture of 3:1 chloroform and methanol (Sigma); and 0%, 1% and 10% w/v of HAp nanoparticles (diameter ≤ 200 nm, Sigma) were homogeneously mixed into this solution. A high-voltage power supply (Gamma High Voltage) applied 18–21 kV on a blunt-tip catheter positioned 4–4.5” from a grounded collector. The polymer solution was fed to the catheter tip by a syringe pump (Kent Scientific) at 1.8–2.1 mL/h and the nanofiber scaffolds were deposited on the grounded collector.

In order to examine the morphology of the nanofiber scaffolds, they were sputter-coated with 10 nm of gold and imaged under high magnification using a field-emission scanning electron microscope (SEM, JEOL JSM-6500F) followed by an EDX spatial elemental mapping examination. Instrument aperture and probe current were adjusted to give a dead time of 15%–20%. Surfaces were analyzed for 5 min at 5–15 kV and a magnification of 100–5000× to provide a complete profile of the different elements present. Spatial element mapping was performed by grouping pixels with similar atomic spectra. Fiber diameters were measured using SEM image analysis software. Ten measurements were made on each scaffold with n_min_ = 30 and size distribution histogram was plotted.

Thermal characterization of the nanofiber scaffolds was performed to determine the effect of electrospinning process and HAp content on polymer crystallinity and thermal stability. Digital scanning calorimetry (DSC, TA Instruments DSC 2920) was used to determine the polymer crystallinity in different nanofiber scaffolds. The scaffolds were heated from 5 °C to 120 °C at 5 °C/min and the crystallinity of a sampled was calculated by the following Equation:

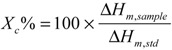
(1)
where H_m,sample_ is the enthalpy of melting of the nanofiber scaffold and H_m,std_ is the enthalpy of melting of 100% crystalline PCL (ΔH_m,std_ = 139 J/g) [39]. Thermogravimetric analysis (TGA, TA Instruments TGA 2950) was used to measure the change in mass as a function of temperature. The nanofiber scaffolds were heated from 25 °C to 700 °C at 10 °C/min and the weight loss was measured between 200 °C and 500 °C. In both the thermal analysis techniques, polymer pellets that had not been subjected to electrospinning process were used as controls, and are noted as “Source PCL”.

### 3.2. Rat Marrow Stromal Cell Culture

MSCs were isolated from male Wistar rats (*Rattus norvegicus*) supplied by Harlan Sprague Dawley, Inc. Limbs were aseptically removed from recently euthanized animals. Soft tissue was removed and the bones were briefly stored in cold PBS before isolating cells. Metaphyseal ends of the bones were removed to expose the bone marrow cavity. In a 50 mL conical tube, marrow was repeatedly flushed with maintenance media (α-MEM with 10% fetal bovine serum (FBS, Sigma) and 1% penicillin/streptomycin (pen/strep, Sigma)) using 10 mL syringes with 18 and 25 gauge needles. Media containing cells and debris was filtered with a 70 μm nylon filter into a clean tube. Cells were counted using a hemocytometer before seeding. All scaffolds were sterilized by exposing them to UV light for 60 min followed while submerged in 70% ethanol. The substrates were then washed twice with warm PBS followed by warm culture media prior to MSC seeding. Cells were seeded on scaffolds in a 24-well plate at a density of 1 million per well. Cultures were incubated at 37.0 °C and 5% CO_2_ for the duration of the study. Half of the media was changed at day 4. On Day 7, the media was replaced with osteogenic differentiation media (α-MEM with 10% fetal bovine serum, 1% penicillin-streptomycin, 10^−8^ M Dexamethosone, 50 mg/mL ascorbic acid, 8 mM β-glycerolphosphate). Media was changed every 2 days for up to 3 weeks of culture. All scaffolds were cultured and assayed in triplicate at each time point specified (*i.e*., 1, 4, or 7 days post-seeding, and 1, 2, or 3 weeks post-differentiation, n = 6).

### 3.3. MSC Adhesion and Proliferation on HAp/PCL Composite Nanofiber Scaffolds

After one, four, and seven days of culture in maintenance media, cell responses to the scaffolds were investigated through cell adhesion. Cells adhesion was investigated using the live cell stain Calcein AM (Invitrogen) (excitation: 485 nm, emission: 530 nm). Calcein AM can penetrate live cell membranes, where the AM is cleaved and the resulting calcein molecule fluoresces green. The cells were incubated in 2 mM of Calcein AM in PBS for 45 min and were imaged with a fluorescence microscope (Zeiss) with appropriate filters. Images were analyzed (ImageJ, NIH) to compute the percent area covered by live cells for comparison with the cell viability assay.

Cell viability was measured after one and four days of culture (during the log phase growth) using a commercially available MTT assay kit (Sigma). Adhered cells were incubated at 37 °C for 3 h in a (3-[4,5-dimethylthiazol-2-yl]-2,5-diphenyl tetrazolium bromide (MTT) solution. Mitochondrial dehydrogenases of viable cells cleave the tetrazolium ring, yielding purple formazan crystals. Formazan crystals were then dissolved in the MTT solvent with 10% (volume) Triton-X. The optical density (OD) of the solvent is proportional to the mitochondrial activity of the cells on the surface. OD was measured at 570 nm using a spectrophotometer (FLUOstar Omega; BMG Labtech, Durham NC). Background absorbance at 690 nm was subtracted from the measured absorbance. 

### 3.4. Osteogenic Differentiation of MSCs on HAp/PCL Composite Nanofiber Scaffolds

MSC responses to the nanofiber scaffolds were investigated after providing the cells with osteogenic differentiation media for one, two, and three weeks. A colorimetric alkaline phosphatase (ALP) assay, total protein assay, quantitative polymerase chain reaction (qPCR), and immunofluorescent staining were used to evaluate the cell responses to nanoarchitecture containing different concentrations of HAp. 

In order to quantify intracellular ALP production after one, two, and three weeks in osteogenic conditions, the cells were lysed in CellLytic^®^ solution (Sigma). ALP activity was measured using a commercially available colorimetric assay kit (Quantichrome™ BioAssay Systems). Briefly, ALP catalyzes the reaction removing the phosphate from *p*-nitrophenolphosphate (*p*-NPP), thus yielding *p*-nitrophenol, and the p-nitrophenol concentration is measured by the absorbance at 405 nm. The same lysate was also used to determine the total intracellular protein content using a commercially available BCA (bicinchoninic acid) assay (Pierce Biotechnology). The absorbance of the solution was measured using a plate reader at a wavelength of 570 nm and was converted to protein content using an albumin standard curve. All the ALP data was normalized with the total protein content to account for changes in number of cells present on each scaffold surface. 

The expression levels of four key bone-related genes were measured with qPCR after one, two and three weeks of culture in osteogenic conditions. Messenger RNA (mRNA) was purified using an RNeasy extraction kit (Qiagen). Genomic DNA (gDNA) contamination was avoided by degrading any remaining gDNA with DNaseI (Fermentas). Complimentary DNA (cDNA) template was generated from mRNA with a first-strand synthesis kit (Fermentas), and both the DNaseI and reverse transcriptase enzymes were thermally inactivated after their respective steps according to the manufacturer’s protocols. cDNA was stored in at −80 °C until further use. For PCR reactions, primers were either designed as documented below, or were purchased as a forward-reverse primer mix (Qiagen). Casein Kinase II (CKII), Ribosomal protein L13A (RPL13A), and RhoA were measured with Qiagen primer sets, while osteopontin (OP) and collagen I (ColI) were measured with custom-designed primers (Table 1). Custom designed primers were validated by running gel electrophoresis the PCR product to ensure that the amplicon length matched the predicted length, and by performing a melt curve step at the end of real-time quantitative PCR (qPCR) to verify the presence of a single amplicon. All four genes were normalized with respect to the housekeeping gene-RPL13A expression levels.

**Table 1:** Custom primers used for qPCR.

Gene	Forward primer	Reverse primer	Amplicon length
Osteopontin	atcaggacagcaacgggaagac	gagttccaaagccagcctggaa	224 bp
Collagen I	acagaggcataaagggtcatcg	cctggcaaagatggactcaacg	159 bp

The amplicon from successful reactions was purified using the ethanol precipitation method [89]. Finally, purified amplicon concentrations were measured with a spectrophotometer (Nanodrop, Thermo Scientific), and standards were then prepared with DNase-free water for use in calculating the copy number from test qPCR reactions.

After three weeks in osteogenic media, the scaffolds were removed and immuno-labeled for osteopontin and osteocalcin. Cells were fixed with 3.7% paraformaldehyde in PBS solution and permeabilized with a 1% Triton-X in PBS solution. A blocking serum of 40 g/mL of Trypan blue (Sigma) and 100 g/mL of bovine serum albumin in PBS was used to reduce non-specific antibody binding. After rinsing and blocking, the scaffolds were incubated in either an osteopontin primary antibody (1:100 in PBS, V-19 purified goat polyclonal antibody of mouse origin, Santa Cruz Biotechnology) or an osteocalcin primary antibody (P-18 purified goat polyclonal antibody of mouse origin, Santa Cruz Biotechnology) for one hour. After an additional blocking step and PBS wash, the scaffolds were then incubated in a FITC-conjugated or TRITC-conjugated secondary antibody (1:200 donkey anti-goat IgG, Santa Cruz Biotechnology) for 45 min in the dark. The scaffolds were rinsed once more before being imaged under 470 nm excitation wavelength using a fluorescence microscope (Zeiss).

### 3.5. Statistical Analysis

All test and control substrates were cultured and assayed in triplicate at each time point specified. The experiments were conducted in duplicate using different animals as the MSC source for each study. All the statistics presented here as a mean +/− standard deviation. A one-way ANOVA with a Tukey adjustment for multiple comparisons was used to determine the effects of treatment (HAp concentration), time (days or weeks where noted), and the interaction of treatment by time (Hap*time). Any effect (HAp, time, or Hap*time) with a p-value less than 0.05 was considered significant and for borderline significant values of 0.05 < p < 0.1 the result was simply noted. Additionally, assay values at each time point were compared using a two-way t-test and significant p-values (p ≤ 0.05) were noted with a star on the corresponding chart.

## 4. Conclusions

PCL nanofiber scaffolds were fabricated to be HAp-free (0% HAp, control), or have either 1% or 10% HAp nanoparticles within the PCL nanofibers. Material characterization methods verified that the desired nanofibrous morphology and compositions were achieved, and a difference in mean fiber diameter was unlikely to significantly affect the cell population. There were difference in the initial levels of metabolic activity, but by day seven, the cell populations, as measured by coverage, were very similar. Once in osteogenic media, ALP activity showed consistencies with previous reports by having elevated levels on 10% HAp scaffolds at the third week. Using qPCR, expression levels for three key genes were measured to be differentially regulated as a result of HAp nanoparticles in a concentration-dependent manner. This was the first study to present time-course qPCR data for key osteoblast-related genes and the information demonstrated high sensitivity to HAp nanoparticles. Future studies will investigate activation for RhoA and CKII as well as potential mechanisms sensing the extracellular chemistry and mechanical environment. 
